# Determination of the internal loads experienced by proximal phalanx fracture fixations during rehabilitation exercises

**DOI:** 10.3389/fbioe.2024.1388399

**Published:** 2024-09-02

**Authors:** Peter Schwarzenberg, Thomas Colding-Rasmussen, Daniel J. Hutchinson, Jorge San Jacinto Garcia, Viktor Granskog, Michael Mørk Petersen, Tatjana Pastor, Tine Weis, Michael Malkoch, Christian Nai En Tierp-Wong, Peter Varga

**Affiliations:** ^1^ AO Research Institute Davos, Davos, Switzerland; ^2^ Department of Orthopedic Surgery, Hvidovre University Hospital, Copenhagen, Denmark; ^3^ Department of Orthopedic Surgery, Rigshospitalet, Copenhagen University Hospital, Copenhagen, Denmark; ^4^ Department of Fibre and Polymer Technology, KTH Royal Institute of Technology, Stockholm, Sweden; ^5^ Biomedical Bonding AB, Stockholm, Sweden; ^6^ Department of Clinical Medicine, Faculty of Health and Medical Sciences, University of Copenhagen, Copenhagen, Denmark; ^7^ Department for Plastic and Hand Surgery, Inselspital University Hospital Bern, University of Bern, Bern, Switzerland

**Keywords:** non-contact measurement, customizable osteosythesis, patient-specific treatment, finite element modeling, CT derived models

## Abstract

Phalangeal fractures are common, particularly in younger patients, leading to a large economic burden due to higher incident rates among patients of working age. In traumatic cases where the fracture may be unstable, plate fixation has grown in popularity due to its greater construct rigidity. However, these metal plates have increased reoperation rates due to inflammation of the surrounding soft tissue. To overcome these challenges, a novel osteosynthesis platform, AdhFix, has been developed. This method uses a light-curable polymer that can be shaped *in situ* around traditional metal screws to create a plate-like structure that has been shown to not induce soft tissue adhesions. However, to effectively evaluate any novel osteosynthesis device, the biomechanical environment must first be understood. In this study, the internal loads in a phalangeal plate osteosynthesis were measured under simulated rehabilitation exercises. In a human hand cadaver study, a plastic plate with known biomechanical properties was used to fix a 3 mm osteotomy and each finger was fully flexed to mimic traditional rehabilitation exercises. The displacements of the bone fragments were tracked with a stereographic camera system and coupled with specimen specific finite element (FE) models to calculate the internal loads in the osteosynthesis. Following this, AdhFix patches were created and monotonically tested under similar conditions to determine survival of the novel technique. The internal bending moment in the osteosynthesis was 6.78 ± 1.62 Nmm and none of the AdhFix patches failed under the monotonic rehabilitation exercises. This study demonstrates a method to calculate the internal loads on an osteosynthesis device during non-load bearing exercises and that the novel AdhFix solution did not fail under traditional rehabilitation protocols in this controlled setting. Further studies are required prior to clinical application.

## 1 Introduction

Traumatic phalangeal fractures are among the most common upper extremity fractures, particularly in younger patients (Karl et al., 2015; Moura et al., 2022). When compared to other injuries, such as knee, lower limb, hip, and skull injuries, fractures of the hand and phalanges constitute the largest economical cost, primarily due to a high number of fractures occurring among patients of working age, 20–64 years old, resulting in time away from employment (de Putter et al., 2012). Accordingly, optimizing the treatment of phalangeal fractures to improve individual patient outcomes may also have a large economic benefit to both patients and society as a whole.

Non-displaced and stable proximal phalangeal fractures are usually treated conservatively, but surgical fixation is generally advocated when the fracture is unstable, i.e., severely displaced, comminuted or if adjacent phalangeal bone fractures are involved (Kremer et al., 2022; Cheah and Yao, 2016). The goal of phalangeal osteosynthesis is to achieve a biomechanically stable fixation that can withstand subtle forces associated with early rehabilitation exercises while avoiding soft tissue damage to adjacent tendons. This in turn reduces the risk of adhesions and finger stiffness (Logters et al., 2018; Miller et al., 2016; Ataker et al., 2017; Kose et al., 2019). The choice of surgical fixation technique and material is determined by the fracture morphology, skin and soft-tissue status, as well as surgeon preference. The most commonly applied techniques are Kirschner wires (K-wires), screws, and plates (Henry, 2008). Metal plates are usually required to achieve optimal fragment fixation in comminuted phalangeal fractures, while K-wires or screws are applied in simple transverse or oblique fractures (Henry, 2008; Kieseritzky et al., 2020). Plate osteosynthesis techniques have been shown to provide an advantage in biomechanical stability and allow early mobilization while K-wires were found to be less associated with tendon adhesions (Cheah and Yao, 2016; Logters et al., 2018). Joint stiffness and tissue adhesions following hand surgery are a multi-faceted issue that is suspected to be caused by the soft-tissue trauma, the surgical approach, as well as the usage of osteosynthesis materials with sharp edges or prominent screws. Issues such as these lead to a reported reoperation rate of 42% for the use of plate osteosynthesis in phalangeal fractures (von Kieseritzky et al., 2017).

A better understanding of the internal loading acting on osteosyntheses is needed in order to ensure that the mechanical properties of the chosen fixation method meet the loading demands during fracture healing. Previously, attempts have been made to quantify the forces associated with activities of daily living (Fowler and Nicol, 1999; Purves and Berme, 1980). However, these only measure the forces acting externally and infer joint forces, not the loading of an osteosynthesis device. Therefore, there is a need to measure the loading exerted on the osteosynthesis device itself, specifically during rehabilitation exercises, such as fingertip-to-palm extension exercises.

To overcome the challenges associated with fracture management, a novel osteosynthesis platform, (Bonevolent^™^ AdhFix, Biomedical Bonding AB, Stockholm, Sweden) has been developed. The basis of this surgical fixation method is a light-curable, viscous composite, that is placed around and over conventional metal cortex screws, which act as anchorage to the bone fragment, thereby allowing for *in situ* customization and anatomical adaptation (Hutchinson et al., 2021). The composite consists of a high concentration of hydroxyapatite along with triallyl and trithiol triazine-trione monomers, which undergo thiol-ene coupling chemistry to form a crosslinked network upon exposure to high energy visible (HEV) light. When cured and hardened in a desired size and shape, the construct provides a plate like structure with a smooth surface and rounded edges (Hutchinson et al., 2021). This technique has shown promising biomechanical results in an *ex vivo* ovine phalanx study (Schwarzenberg et al., 2023) and has been shown to not induce soft tissue adhesions in an *in vivo* rat femur fracture model (Kieseritzky et al., 2020; Arseneault et al., 2018). AdhFix has the advantage of being shaped *in situ* on the corresponding bone surface as a patient specific plate, which may be a biomechanical advantage providing more three-dimensional support compared to conventional factory-made metal plates (Park et al., 2021). Accordingly, AdhFix might be a clinically viable fracture fixation technique for certain proximal phalanx fractures. The previous *ex vivo* studies have shown that while the ultimate bending strength of AdhFix was lower than conventional metal plating, it may have provided enough biomechanical stability to support common rehabilitation exercises with freshly fractured phalanges (Hutchinson et al., 2021; Schwarzenberg et al., 2023). In addition, the modes of failure of AdhFix and metal plating were very different, with the former failing due to fracturing of the AdhFix fixation patch while the later failed due to catastrophic failure of the bone (Schwarzenberg et al., 2023), which suggested that the plating was stronger than necessary for stabilizing the fracture. However, this remains unknown due to a lack of knowledge on the loads that the osteosynthesis needs to withstand.

The primary aim of this study was to measure the bending loads occurring within human midshaft proximal phalanx fracture fixations during simulated rehabilitation exercises, namely the fingertip to palm articulation which was achieved by pulling on the profundus flexor tendon of each finger. The internal bending loads were determined through measuring the displacement of a PEEK plate across an ostectomy during the finger flexing exercise. Furthermore, since this study was a first of its kind, as a proof of concept and to put the study into context, validation tests were performed to evaluate whether AdhFix was stable enough to withstand these loads by monotonically subjecting AdhFix fixations to the same protocol.

## 2 Methods

### 2.1 Overview

This study is divided into two parts. Part 1 investigates the internal loads applied to plate osteosyntheses using a novel testing method in the proximal phalanx under simulated rehabilitation exercises. To mimic rehabilitation exercises, each finger was fully flexed via the profundus flexor tendon from a fully extended state until the fingertip touched the palm. The biomechanical concept relies on a plastic (polyether ether ketone, PEEK) plate with known biomechanical properties placed over a 3 mm fracture gap and optical markers fixed to each bone fragment allowing for the calculation of the internal loads applied when deformation was analyzed with a stereographic camera system and an FE model. The gap osteotomy was required to simplify the loading condition and prevent any confounding far cortical contact. In part 2, as a proof of concept, and to put the experiment in context, the AdhFix osteosynthesis technique was applied in a human *ex vivo* proximal phalanx midshaft transverse fracture model with a reduced fracture and a 3 mm fracture gap. Accordingly, the following methods sections describe the application and analysis of the internal loads, followed by the testing of the AdhFix osteosynthesis technique. Additionally, AdhFix was tested during a grip-based motion until failure of the AdhFix patch to mimic activities of daily living. Accordingly, this study examines the internal loads occurring in a proximal phalanx fracture during full finger flexion via PEEK-plate bending and FE-analyses and subsequently investigates if these loads can be withstood by the AdhFix fixation technique.

### 2.2 Specimen information

Five human cadaver arms (above elbow) were used (Table 1). The donors were all male, had an average age of 41.8 ± 6.0 years, weight of 86.5 ± 5.7 kg, and height of 179.3 ± 8.6 cm. Second, third, and fourth proximal phalanges were used for biomechanical analysis in each specimen. Four specimens were used in measuring the internal loads in the osteosynthesis (Specimens 1-4, Part 1) while one was reserved for a stable well-reduced fracture model in the proof-of-concept testing with the AdhFix platform (Specimen 5, Part 2) and one hand was reused from the determination of internal loads (Specimen 2) for testing AdhFix with an unstable fracture (Part 2). All donors gave their informed consent inherent within the donation of the anatomical gift statement during their lifetime and were screened for bone health, neurological diseases such as Parkinson due to stiffness of the joints, bipolar disorder or epilepsy due to unknown/undocumented injuries, and previous orthopedic trauma of the hand. Prior to all experimental procedures, a high-resolution peripheral quantitative computer tomography (HR-pQCT, XtremeCT; Scanco Medical AG, Brüttisellen, Switzerland) scan was performed on each specimen. Each scan had an X-ray voltage of 60 kVp, X-ray current of 0.90 mA, and an isotropic voxel size of 82 µm. This scan provided a quantitative measurement of bone geometry and bone mineral density of the intact phalanges.

**TABLE 1:** Cadaver specimen and donor information.

Specimen Id [--]	Side [L/R]	Use case [Loading/AdhFix]	Gap size [mm]	Sex [M/F]	Age [years]	Height [cm]	Weight [kg]	BMI [-]
Specimen 1	Right	Loading	3	Male	37	193	88.9	24.6
Specimen 2	Left	Loading and AdhFix	3	Male	41	170.2	82.1	28.4
Specimen 3	Right	Loading	3	Male	50	180.3	95.3	29.3
Specimen 4	Right	Loading	3	Male	47	170.2	78.9	27.3
Specimen 5	Right	AdhFix	0	Male	34	182.9	87.1	26.5
				Average	41.8	179.3	86.5	27.2
				Std	6.0	8.6	5.7	1.6

### 2.3 Surgical procedure

**FIGURE 1:**
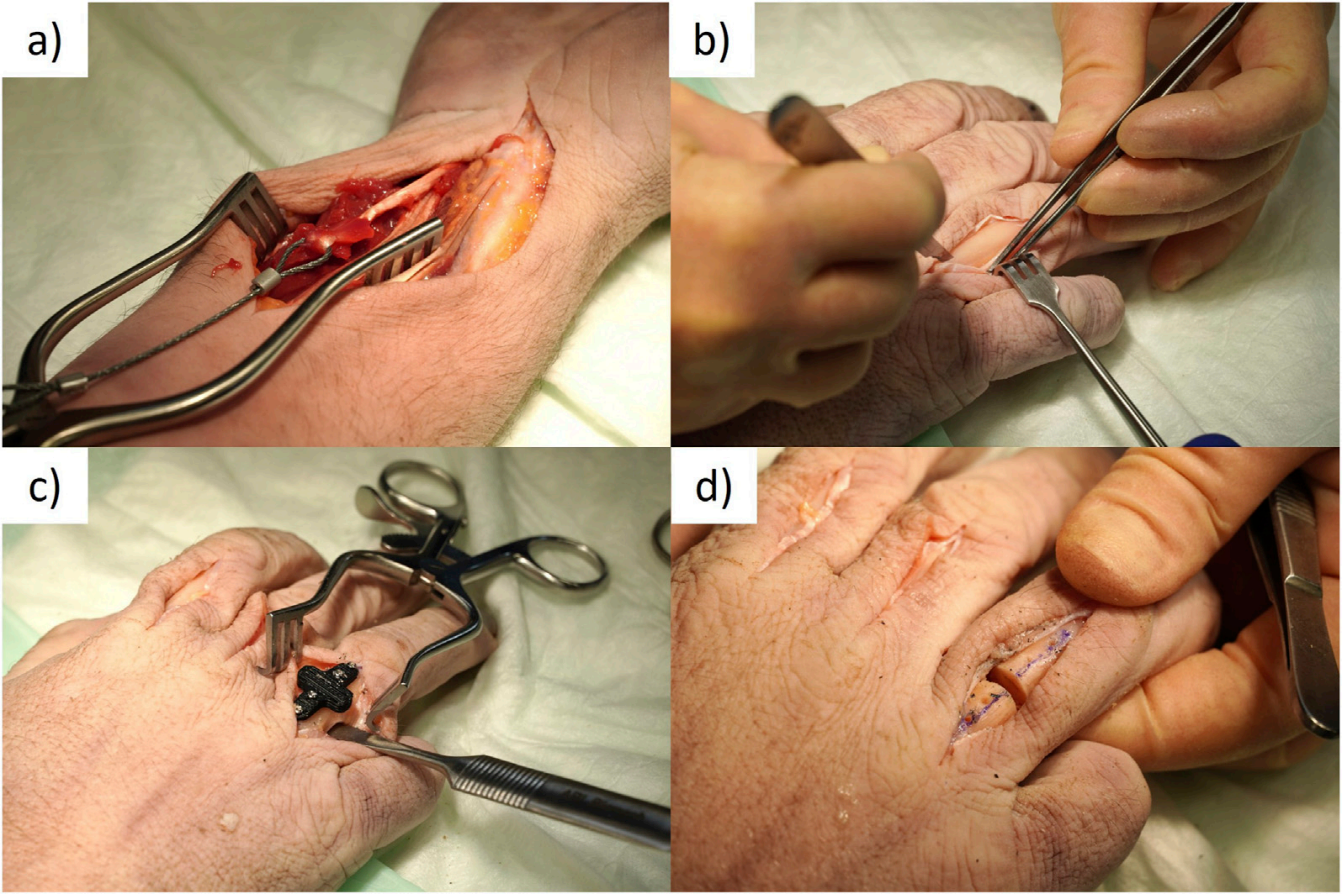
Surgical procedure. **(A)** Braided steel cable attached to the flexor tendon with sutures. **(B)** Exposed proximal phalanx through extensor tendon split with periosteum separated to access the bone. **(C)** Osteotomy guide attached to phalanx with cortical screws and blunt retractor protecting tendons on volar side of the hand. **(D)** Osteotomized phalanx.

For specimens 1-4 designated to test the internal loading in the osteosynthesis device (Part 1), the flexor digitorum profundus tendon for each of the second, third, and fourth digits was harvested through a longitudinal midline incision approximately 4 cm proximal to the distal wrist crease to avoid damage to the flexor retinaculum. Each tendon was attached to a 1.5 mm braided steal wire through a loop and attached with conventional running nylon sutures (Figure 1A).

Next, the proximal phalanges of the second, third, and fourth finger of each specimen were exposed through a dorsal longitudinal skin incision. The extensor tendon was split through the center with a scalpel and the opening was maintained with a self-retaining retractor (Figure 1B). An incision was made in the periosteum of the proximal phalanx, and it was carefully separated to provide access to the bone. Each phalanx was then osteotomized with a 3 mm gap at the mid diaphysis. A custom 3D printed drilling and cutting guide was employed for this task. The guide was designed to be small enough to fit within the soft tissue incision. It had four screw holes, two on either side of the fracture, spaced 5 mm apart and 5 mm from the fracture line. The guide was held to the bone surface with surgical clamps and unicortical pilot holes were drilled in the near cortex with a 1.1 mm drill bit (DePuy Synthes, Zuchwil, Switzerland). Cortical screws (1.5 mm, DePuy Synthes) were inserted to fix the cutting guide in place. The depths of the pilot holes were measured using a depth gauge and the cortical screws were cut such that they had unicortical attachment. Next, to ensure the safety of the tendons, a blunt retractor was placed between the bone and the soft tissues on the volar side through the dorsal access (Figure 1C). Using a 0.6 mm thick oscillating bone saw, a 3 mm gap osteotomy was cut in the diaphysis of the bone through the slots in the cutting guide (Figure 1D).

**FIGURE 2:**
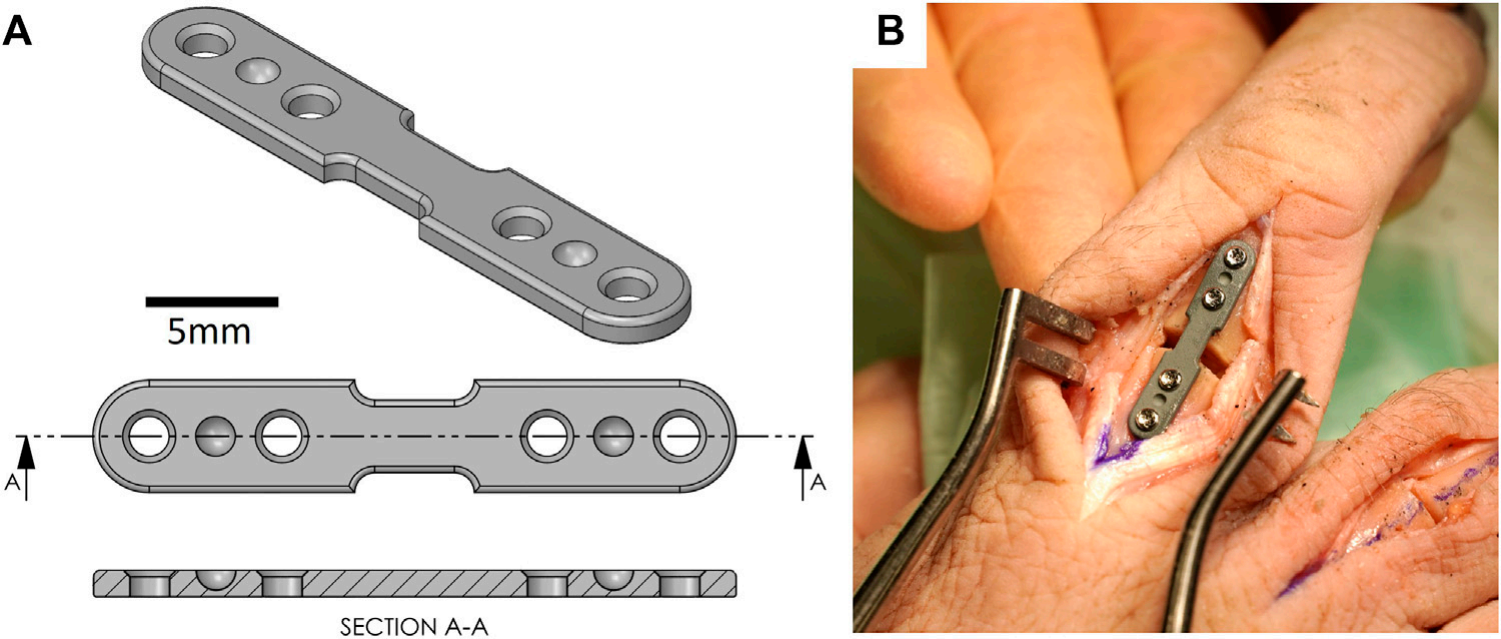
**(A)** Computer aided design (CAD) model of PEEK plate. **(B)** Representative picture of PEEK plate instrumented on a cadaveric proximal phalanx.

After the osteotomy, a custom PEEK plate was used to fix the osteotomy with four 1.5 mm cortex screws (DePuy Synthes) placed in the same holes used to attach the cutting guide (Figure 2). This custom plate was designed based on the dimensions of a clinical-standard stainless steel locking plate (1.5 mm LCP Compact Hand, DePuy Synthes); however, it was de-featured to provide a more predictable bending behavior across the fracture gap. The design and material of this plate were chosen to allow for larger displacements for higher resolution measurements. Furthermore, reference divots were added such that the same points could be identified during mechanical testing and during subsequent computer simulations (Figure 2). Following the surgical procedures, each hand was HR-pQCT-scanned again with the same protocol as above. Prior to scanning, the PEEK plate was coated in a thin layer of zinc paint to allow identification on the scan.

### 2.4 Internal loads measurement biomechanical test setup

**FIGURE 3:**
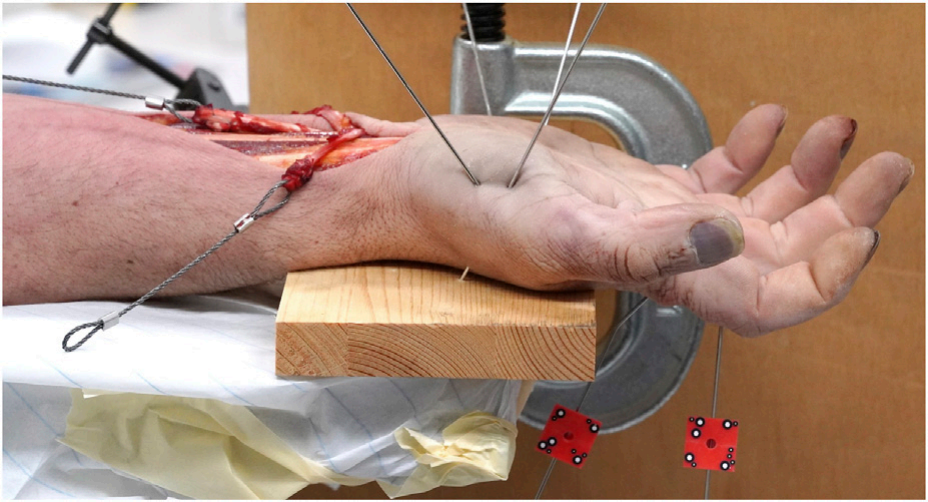
Mechanical test setup showing the hand specimen fixated to the wooden substrate, the steel cable connecting the tendon to the testing machine via a pulley and the red optimal markers attached to each bone fragment via K-wires.

After scanning, each hand was securely fixed to a wooded board with the palm oriented upwards (Figure 3). This fixation was achieved by placing two K-wires through both the first and fifth metacarpals into the wooden board. The wooden board was then rigidly fixed to the base of an electrodynamic testing machine equipped with a 3 kN load cell (Acumen, MTS, Eden Prairie, MN, United States). The aforementioned braided steel cables that were attached to the flexion tendons were then attached to the testing machine though a pulley such that the vertical load of the machine could be translated into a force vector along the axis of the forearm. The axial translation of the machine and the accompanying force were measured. 1.6 mm K-wires were placed on both the proximal and distal bone fragments, just external to the PEEK plate. Optical markers were then fixed to these K-wires to measure the relative displacement between the fragments with a stereographic camera system (Aramis SRX, GOM GmbH, Braunschweig, Germany) over the course of flexion. Using a touch probe, the reference divots described earlier on the PEEK plate were probed and the motions of the bone fragments were calculated at the position of these divots by the camera system’s software.

Rehabilitation exercises were simulated by fully extending the hand as the starting position and then individually flexing each digit until the fingertip touched the palm by pulling on the flexor profundus tendon. The actuator of the testing machine pulled the tendons at a rate of 1 mm/s and tests were repeated three times for each digit. These tests were monotonic in nature.

### 2.5 Finite element modeling

In Part 1, the HR-pQCT scans were used to build specimen specific 3D models of each phalanx in Amira 3D (v2021, Thermo Fisher Scientific, Hillsboro, OR, United States). The post-op scan was imported, and the plate was manually aligned to a computer aided design (CAD) model of a plate oriented at the coordinate system origin. Next, the pre-op scan was imported and segmented with density bounds of 400–4000 HU (Schwarzenberg et al., 2021). Each bone fragment mask was then smoothed and filled. The segmented image data was then spatially co-registered to the post-op scans using a normalized mutual information metric. Finally, the transformed bone fragments from the pre-op scan were cropped to match the osteotomy pattern of the post-op scans. This created artifact free geometry and image data of the bone fragments in the post-op orientations. The masks and image data were exported for geometry and density data, respectively. This process was repeated for the second, third, and fourth digits on Specimens 1 - 4.

These data sets were then imported into Simpleware (v17, Simpleware Ltd., Exeter, United Kingdom) where they were meshed with quadratic tetrahedral elements for use in finite element modeling. A mesh convergence study found less than 5% difference in the parameters of interest between mesh sizes with the following final settings: minimum edge length of 0.25 mm, a maximum error of 0.082 mm, and a maximum edge length of 1 mm with 0.25 mm maximum edge length in a refinement area near the plate. CT-based material properties were assigned to each bone element based on a previously published scaling law (Schwarzenberg et al., 2019; Dailey et al., 2019) using the local bone mineral density values from the pre-OP HR-pQCT scan.

**FIGURE 4:**
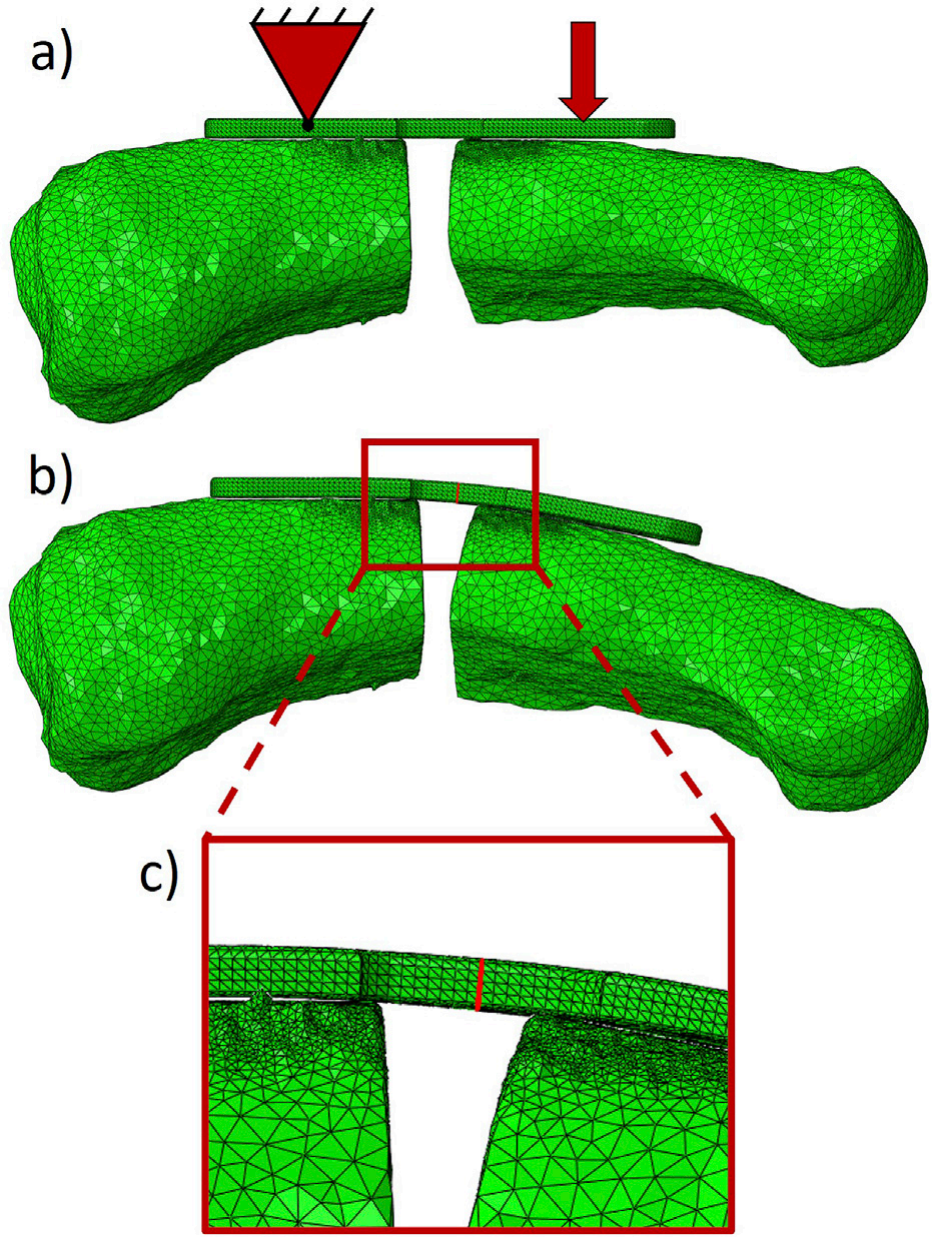
Finite element model of a representative phalanx with **(A)** boundary conditions of a pin support and applied displacement show in red. **(B)** The same model is shown with calculated displacements with cut plane at center of the plate shown as a red line. **(C)** Zoomed-in view of region of interest highlighting cut plane of plate.

The meshed bone fragments were then imported into Abaqus CAE (v2021, Dassault Systems, Velizy-Villacoublay, France). Additionally, the CAD model of the PEEK plate was also imported into Abaqus. The plate was meshed in Abaqus CAE with quadratic tetrahedral elements to a global size of 0.25 mm based on the same mesh convergence study mentioned above (Figure 4A). Material properties of PEEK were determined via uniaxial tension testing of dog bone shaped specimens (N = 5), providing an average Young’s modulus of 1740.5 MPa, a Poisson’s ratio of 0.3779, and a yield stress of 100.3 MPa for numerical application. The dimensions of the dog bone samples were 63.5 mm in length, 9.53 mm in width, and 4 mm thick. The gauge length of the test region was 9.53 with a width of 3.18 mm, with grip regions of 18.58 mm. Additionally, the detailed post-elastic yield stress – plastic strain relationship was measured. To simulate the effect of screws, the screw holes in the plate were tied to the nodes on the surface of the bone directly underneath. A frictionless contact condition was implemented between the bottom surface of the plate and the bone fragments as well as between the cut surfaces of the bone at the fracture gap. Displacement boundary conditions were applied by fixing all six degrees of freedom on the proximal bone fragment reference divot of the plate and imposing the experimentally assessed transformations measured by the camera system on the distal fragment in 10 evenly spaced time steps. Non-linear geometrical effects were considered in the simulations (Figure 4B). The resulting bending moment was calculated at the center of the plate in a cut plane that transformed with the deformation of the loading to remain tangent at the centerline (Figures 4B, C).

### 2.6 AdhFix biomechanical testing

**FIGURE 5:**
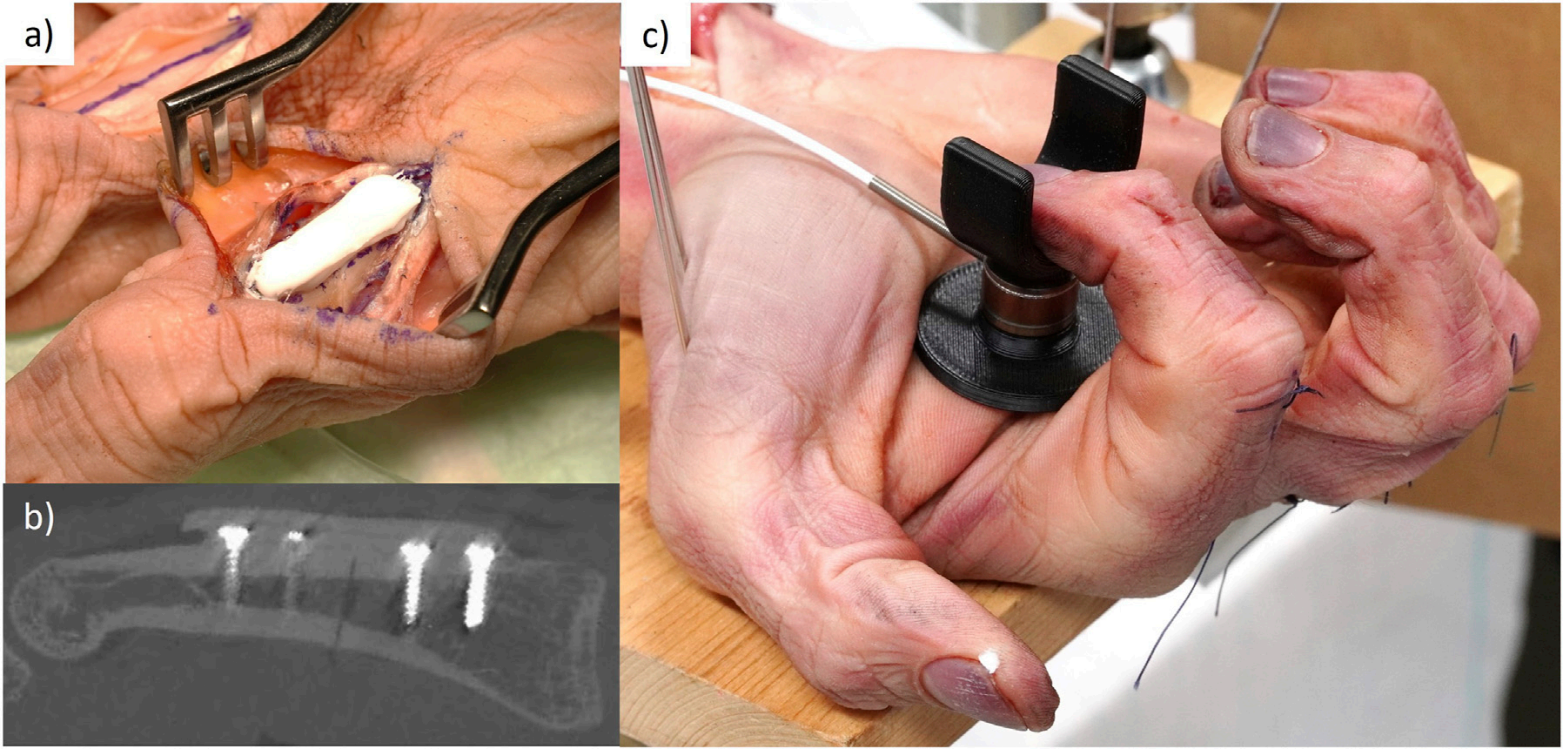
**(A)** Representative picture of an AdhFix patch applied to the proximal phalanx. **(B)** slice view from CT scan of the AdhFix patch after application. **(C)** Testing set up of the AdhFix patches to failure with loadcell seen in black.

After the determination of the internal loads, the AdhFix platform was applied to two specimens in order to test if the osteosynthesis could withstand the bending forces associated with the fingertip-to-palm exercises (Part 2). The specimens used were the reserved intact cadaver specimen (Specimen 5), where the same procedure as in Section 2.3 was applied with a well-reduced simple midline proximal phalanx fracture, and a reused (Specimen 2) cadaver specimen with a 3 mm fracture gap maintained by a custom 3D-printed spacer. AdhFix was applied according to a previously described protocol (Colding-Rasmussen et al., 2023). The AdhFix composite consists of triallyl and trithiol triazine-trione monomers, together with a photo-initiator, diphenyl(2,4,6-trimethylbenzoyl)phosphine oxide) (TPO), and hydroxyapatite microparticles. Upon curing through HEV light induced thiol-ene coupling chemistry, the composite has a reported flexural modulus of 6.6 ± 0.2 GPa and a flexural strength of 69 ± 3 MPa (Hutchinson et al., 2021). The fluid composite was first placed around all four screw heads, which were tightened “two-finger-tight,” after which the composite was cured with a HEV light source (Bluephase PowerCure LED lamp, Ivoclar Vivadent Clinical, Schaan Liechtenstein). Subsequently, two more layers of composite with curing in-between were applied making a total osteosynthesis plate of approximately 25 mm × 6 mm × 3 mm in length, width and thickness, respectively (Figure 5A). After the osteosynthesis was complete, the periosteum, tendon sheet, and skin were closed using conventional running sutures. This was repeated for the second, third, and fourth phalanx on both hands.

CT scans were repeated with the same settings as in Section 2.2 on the specimens with completed AdhFix patches. The thickness of the AdhFix patch was measured at the fracture site from the scans (Figure 5B).

Biomechanical testing was performed using the same setup outlined in Section 2.4. First, each digit was pulled to full flexion (fingertip to palm) three times each, to evaluate if the AdhFix platform could withstand the same rehabilitation protocol. Next, a calibrated force transducer (1 kN, Strain Measurement Devices, Chedburgh, England, S402 122-6604-444) was placed on the palm above the metacarpophalangeal joint of each digit and the tendon was pulled until the fingertip touched the sensor (Figure 5C). The particular digit was then flexed via the flexor tendon pulley mechanism until failure of the fixation patch and the corresponding force was measured.

### 2.7 Statistics

Mean and standard deviation were calculated in Excel and all results are reported as such unless otherwise stated. One-Way ANOVAs were performed in SPSS 27 (IBM Corp. Armonk, NY, United States) to determine group differences between the maximum bending moment calculated in the different hands and digits. Statistical significance was determined at a level of *p* < 0.05.

## 3 Results

**FIGURE 6:**
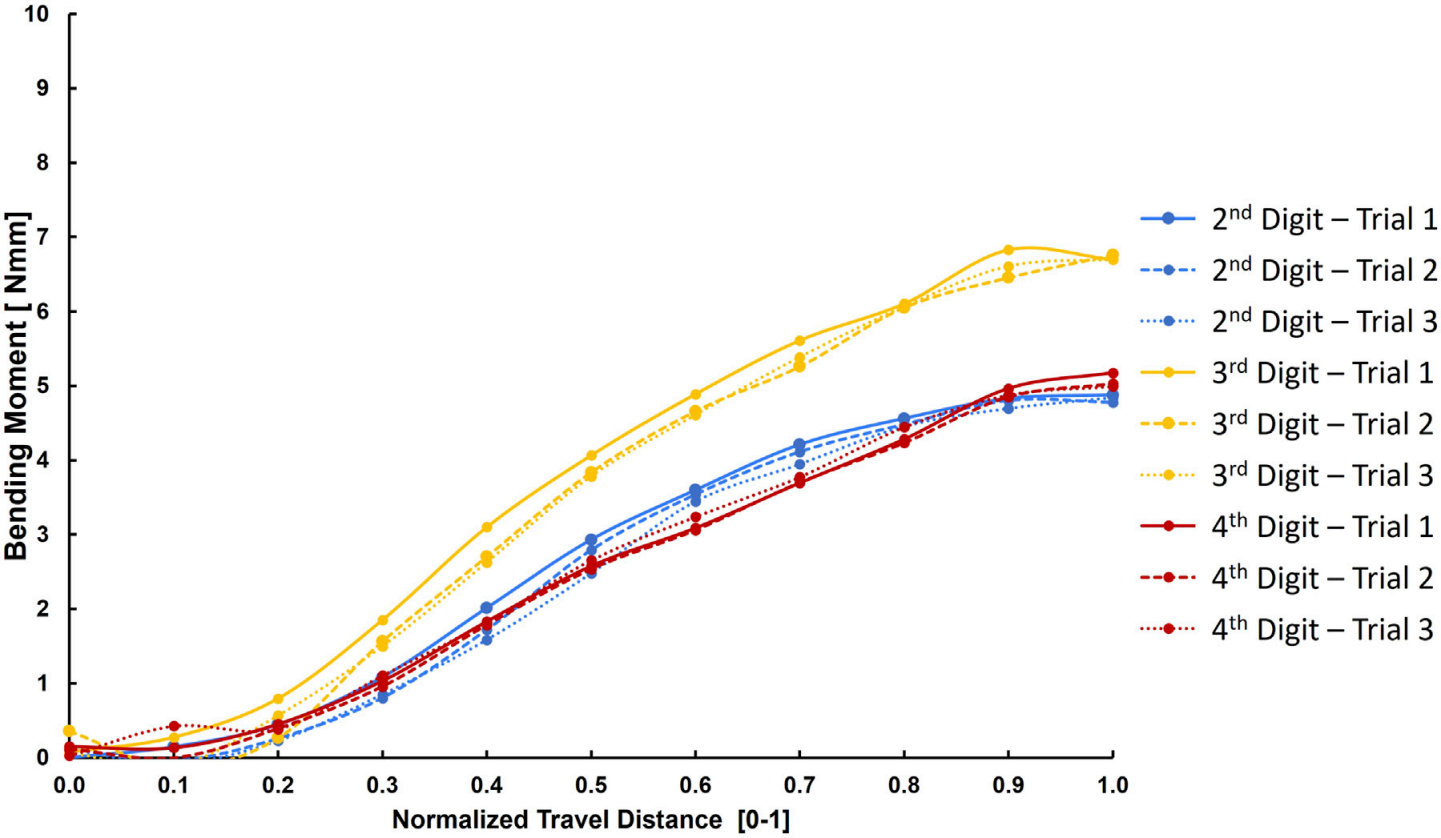
Exemplary figure of the calculated bending moment across the 10 evenly spaced time steps.

**FIGURE 7:**
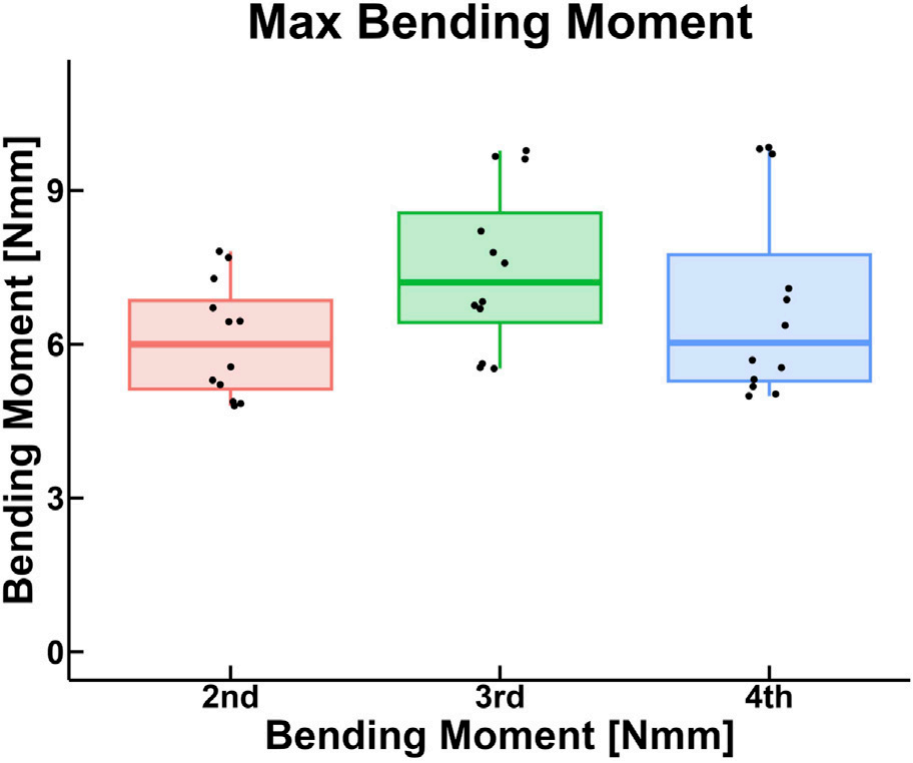
Box plot of the maximum bending moment calculated in the osteosynthesis, grouped by digit.

In part 1, the resulting bending moment from the 10 evenly spaced time steps can be seen in an exemplary figure showing each trial for each digit (Figure 6). The maximum bending moment was calculated as the maximum value on each of these curves. The internal bending moment in the osteosynthesis, as calculated by finite element analysis, was 6.78 ± 1.62 Nmm and did not differ between specimens or fingers (*p* > 0.05) (Table 2; Figure 7). When testing for normality in the maximum bending moment with a Shapiro-Wilk test, two groups by specimens (*p* = 0.003 and 0.009) and one group by finger (*p* = 0.009) were not normally distributed. However, the one-way ANOVA test is robust to deviations from the normal and was used regardless (Maxwell, 2004). Homogeneity of variance was met as assessed by Levene’s test of equality of variances based on median for both groups.

**TABLE 2:** Internal bending moments in osteosynthesis during finger-to-palm rehabilitation exercises.

Phalanx max bending moment during rehabilitation				
Donor	2nd [Nmm]	3rd [Nmm]	4th [Nmm]	By specimen
Specimen 1	5.36 ± 0.15	9.68 ± 0.07	6.78 ± 0.3	7.27 ± 1.81
Specimen 2	7.6 ± 0.23	7.86 ± 0.26	5.52 ± 0.15	6.99 ± 1.07
Specimen 3	4.84 ± 0.03	6.76 ± 0.05	5.07 ± 0.08	5.56 ± 0.86
Specimen 4	6.53 ± 0.12	5.56 ± 0.04	9.79 ± 0.06	7.3 ± 1.81
By finger	6.08 ± 1.08	7.47 ± 1.52	6.79 ± 1.85	6.78 ± 1.62

In part 2, when testing the AdhFix patches in the cadaver samples, all AdhFix samples in both groups, 0 and 3 mm gap fractures, endured the fingertip to palm motion 3 times without failure. When testing the ultimate flexion until failure using the force transducer, the AdhFix platform failed at a mean of 48.48 N (range: 27.4–61.1) in the 0 mm gap group and at 37.78 N (range: 29.4–49.6) in the 3 mm gap group (Table 3).

**TABLE 3:** Failure loads of the AdhFix patches during the ultimate flexion until failure tests.

Hand	Gap	Digit	Load cell force [N]	Patch thickness [mm]
Hand #, S22	0 mm	Second	61.1	4.0
		Third	56.9	3.1
		Fourth	27.4	3.1
Hand #, C22	3 mm	Second	34.3	3.1
		Third	29.4	3.4
		Fourth	49.6	2.9

## 4 Discussion

This is the first study to shed light on the previously unknown magnitude of internal loads that act on the osteosynthesis of midline proximal phalanx fractures. Using a novel combined experimental and computational approach, we found that during a simulated rehabilitation exercise, an average bending moment of 6.78 ± 1.62 Nmm was applied to the osteosyntheses during full finger flexion. Additionally, using this biomechanical setup, we found that the AdhFix patches endured this load in full fingertip to palm motion without failure and that AdhFix failed at a range of 27.4–61.1 N in the reduced fracture group and 29.4–49.6 N in the 3 mm gap group, as measured from the palmar forced grip transducer. Some of this variation could be explained by the thickness of the AdhFix patch, with the thickest patch in the reduced fracture sustaining the highest forces; however, this trend did not persist in the 3 mm gap fracture. The 3 mm gap fracture is designed to represent a comminuted, unstable scenario, leading to more variables that could affect the load at failure.

Biomechanical testing serves as a fundamental tool in orthopedics, particularly for comparing and evaluating the clinical potential of novel techniques and osteosynthesis platforms. Standard analyses, such as static bending, torsion, compression, and tension, along with cyclic testing, are employed across various models including synthetic bone, animal, and human cadavers. However, to our knowledge, an evaluation of internal loads as presented in this study has not before been published (Augat et al., 2021; Schorler et al., 2017; Schorler et al., 2018). This approach might be a valuable addition to conventional biomechanical testing as it determines the actual loads applied to a certain fracture – in this study a phalanx shaft fracture, though the concept could be applied to other fracture types.

Quantifying internal loads during a simulated rehabilitation exercise allows surgeons and researchers to estimate if a current fracture fixation technique will provide sufficient stability to sustain the clinically relevant loads applied during the initial healing period after osteosynthesis. Additionally, such analysis might serve as a guide for future osteosynthesis techniques. Fracture specific fixation platforms could be designed in accordance with such internal force studies, which would show the load the fracture is exposed to during the initial healing period after osteosynthesis and therefore the load the fixation would need to withstand to provide adequate biomechanical stability.

Advancements in computational modeling have significantly enhanced surgeons’ abilities to analyze, predict, and optimize biomechanical performance relative to the fixation of diverse fractures (Lewis et al., 2021). However, the translation from computational models to real-world clinical application introduces additional variables such as pulling forces from surrounding soft tissue as well as complicated modes of mobility that are difficult to capture in detail through computational modelling. These forces could be added through use of such analyses as presented in this current study (Park et al., 2021; Colding-Rasmussen et al., 2023; Brouwer de Koning et al., 2023; Meslier and Shefelbine, 2023).

Additionally, an increased awareness of potential stress shielding and osteosynthesis failure due to over-rigidity and excessive ultimate stability, particularly in osteoporotic fractures, has led to advancements in patient-specific custom fracture fixation platforms (Caiti et al., 2019). The technique presented in this study might be an asset in designing such plates to fit a current fracture type in both anatomy and biomechanical fixation *in vivo* demands.

In a previous study, AdhFix was tested in an *ex vivo* ovine proximal phalanx midshaft 3 mm fracture gap model in 4-point bending at quasi static loading (Schwarzenberg et al., 2023). Here, a mean max bending moment and corresponding standard deviation of 1220 ± 300 Nmm was found with the lowest bending moment to failure being 728 Nmm. Compared to the internal loads applied to a human proximal phalanx midshaft fracture during fingertip to palm as presented in this current study (6.78 ± 1.62 Nmm), AdhFix provided approximately 100 times higher bending stability. Accordingly, though the AdhFix osteosynthesis platform was previously shown to be inferior in max bending moment compared to a conventional metal plate, this study suggests that it could provide sufficient biomechanical strength to stabilize a midshaft proximal phalanx fracture during fingertip-to-palm rehabilitation exercises.

In this study, when testing grip strength of each digit, we found that the minimum load at failure during ultimate flexion of the AdhFix fixation platform of midshaft proximal phalanx fractures was 27.4 N (ring finger) or 29.4 N (middle finger) for the 0 and 3 mm gap fractures, respectively. Ideally, patients would not perform strenuous activities following proximal phalanx osteosynthesis until after a certain rehabilitation period had passed, allowing for the fracture to begin the healing process. In a study evaluating individual finger forces during a variety of activities of daily living assessed by capacity sensors on the tips of the thumb, index, middle and ring fingers, the authors reported force loads in healthy persons (15.9–25.5 N, thumbs excluded) (Fowler and Nicol, 1999), that did not exceed the minimum failure loads as found in this study (27.4 N). Accordingly, this study indicates that AdhFix could provide sufficient biomechanical stability in proximal midshaft phalanx fractures to allow early rehabilitation and simple activities of daily living, which is commonly acknowledged. However, future studies are warranted to determine the performance of such a solution under repetitive cyclic loading as would be expected during the rehabilitation period.

This study is not without limitations. In a cadaver study, there are typically more variations between samples in aspects such as bone morphology and tendon elasticity; however, the cohort used in this study was relatively homogeneous, and this remains an unknown. While cadaver studies can ensure a broader range of variables were tested, they are not under our control and can add variation to the study. Furthermore, when using cadaver specimens, there are ethical and fiscal restrictions and smaller sample sizes, and limited testing groups must be accepted. Due to the availability, all specimens were male; however, this can be viewed as a worst-case scenario for the testing performed. Furthermore, the focus of this study was not the clinical applicability, which can be found in more detail in previous publications (Hutchinson et al., 2021; Colding-Rasmussen et al., 2023). Lastly, as these were cadaver samples, the various muscle forces present during clinical rehabilitation were absent as well as a lack of cyclic loading of the osteosynthesis, as cyclic testing was not feasible with this setup and thus beyond the scope of this work, requiring further investigation.

In summary, this study established a novel approach for the evaluation of internal loads acting on osteosynthesis devices during non-load bearing rehabilitation exercises. The results show that the novel AdhFix solutions can be strong enough to withstand simulated fingertip-to-palm exercises as was the case in this controlled setting. Further studies are required before this technique can be applied in clinical practice.

## Data Availability

The datasets presented in this study can be found in online repositories. The names of the repository/repositories and accession number(s) can be found below: https://zenodo.org/records/10677536.
